# Endophytic *Colletotrichum fructicola* KL19 and Its Derived SeNPs Mitigate Cd-Stress-Associated Damages in *Spinacia oleracea* L.

**DOI:** 10.3390/plants13172359

**Published:** 2024-08-24

**Authors:** Yingxia Wu, Shiru Huang, Wei Tian, Shengyu Yang, Wenshu Shen, Jinyan Dong

**Affiliations:** Key Laboratory of Eco-Environments in Three Gorges Reservoir Region, Ministry of Education, School of Life Sciences, Southwest University, Chongqing 400715, China; zy20210330@email.swu.edu.cn (Y.W.); hsrhsrhsr1@outlook.com (S.H.); twlch1@email.swu.edu.cn (W.T.); yangshengyu@email.swu.edu.cn (S.Y.); shenwenshu@outlook.com (W.S.)

**Keywords:** biogenic SeNPs, endophytic fungus, small-leaf spinach, cadmium, antioxidant activity

## Abstract

The application of nanotechnology in agriculture has received much attention in order to improve crop yield, quality and food safety. In the present study, a Cd-tolerant endophytic fungus *Colletotrichum fructicola* KL19 was first ever reported to produce SeNPs, and the production conditions were optimized using the Box–Behnken design in the Response Surface Methodology (RSM-BBD), achieving a peak yield of 1.06 mM under optimal conditions of 2.62 g/20 mL biomass, 4.56 mM Na_2_SeO_3_, and pH 6.25. Following this, the properties of the biogenic SeNPs were elucidated by using TEM, DLS, and FTIR, in which the 144.8 nm spherical-shaped SeNPs were stabilized by different functional groups with a negative zeta potential of −18.3 mV. Furthermore, strain KL19 and SeNPs (0, 5, 10, 20 and 50 mg/L) were inoculated in the root zone of small-leaf spinach (*Spinacia oleracea* L.) seedlings grown in the soil with 33.74 mg/kg Cd under controlled conditions for seven weeks. Impressively, compared with Cd stress alone, the strain KL19 and 5 mg/L SeNPs treatments significantly (*p* < 0.05) exhibited a reduction in Cd contents (0.62 and 0.50 folds) within the aboveground parts of spinach plants and promoted plants’ growth by improving the leaf count (0.92 and 1.36 folds), fresh weight (2.94 and 3.46 folds), root dry weight (4.00 and 5.60 folds) and root length (0.14 and 0.51 folds), boosting total chlorophyll synthesis (0.38 and 0.45 folds), enhancing antioxidant enzymes (SOD, POD) activities, and reducing the contents of reactive oxygen species (MDA, H_2_O_2_) in small-leaf spinach under Cd stress. Overall, this study revealed that utilizing endophytic fungus *C. fructicola* or its derived SeNPs could mitigate reactive oxygen species generation by enhancing antioxidant enzyme activity as well as diminish the absorption and accumulation of Cd in small-leaf spinach, promoting plant growth under Cd stress.

## 1. Introduction

As industrialization progresses rapidly, the issue of heavy metal contamination has emerged as a ubiquitous environmental concern at the global level, presenting a formidable challenge to vegetation development and the well-being of ecosystems, among which cadmium (Cd) pollution has emerged as one of the most serious ecological problems worldwide [1]. The World Health Organization (WHO) recognized Cd as a priority food contaminant, while the International Agency for Research on Cancer (IARC) classified it as a human carcinogen [2]. Exposure to Cd could have detrimental effects on crops, including a reduction in chlorophyll content, inhibition of photosynthesis, decreased activities of crucial enzymes, and alterations in plant metabolism, ultimately affecting both yield and quality [3]. Beyond this, the increased levels of reactive oxygen species (ROS) generated under Cd stress adversely impact hormone homeostasis, respiration, ion absorption, oxidation–reduction processes, and various other physiological mechanisms within plants [4]. Strikingly, spinach (*Spinacia oleracea* L.) possessed a relatively high capacity for absorbing heavy metals such as Cd [5], and Cd toxicity reduced the antioxidant enzyme activities of spinach, promoting oxidative stress and facilitating Cd absorption through the accumulation of ROS. This toxic effect resulted in a significant decline in biomass, chlorophyll and carotenoid content, photosynthetic rate, transpiration rate, water use efficiency, and substomatal CO_2_ concentration [6]. Green leafy vegetables occupied a pivotal position in the human diet, and their quality held immense importance for the well-being of human health. Therefore, it is of utmost importance to develop and implement efficient, reproducible, and easily scalable eco-safe remediation technologies to mitigate the uptake and accumulation of Cd in edible portions of vegetables, safeguarding human health.

Currently, nanotechnology has shown tremendous potential in addressing heavy metal pollution. The high surface-to-volume ratio and versatile surface properties of nanoparticles (NPs) enhanced their potential for biological activity and reactivity, often serving as nanofertilizers to boost crucial physiological and biochemical functions in plants, effectively reducing the damage from plant stress caused by heavy metals and preventing their accumulation in the food chain [7]. Numerous NPs were synthesized and made progress in their application for preventing heavy metal contamination in staple cereal crops. Specifically, inorganic metal NPs like Zn, Ag, Cu and Se occupied a pivotal position in biological activities and their nano-scale forms [8]. Additionally, microbial NPs synthesis beats chemical/physical methods with advantages like safety, eco-friendliness, and cost-effectiveness [9]. Compared to bacteria, fungi were renowned for their intricate metabolic pathways and remarkable ecological adaptability, which empowered them to thrive and proliferate in a wide range of environmental conditions [10]. It has been demonstrated that fungi had synthesized SeNPs in many conditions [11,12]. By precisely controlling these parameters like fungal growth stage and fermentation conditions, the production process of NPs can be further optimized, resulting in higher yields and superior-quality NPs [13]. Taking these considerations into account, the investigation into the synthesis of NPs using different fungal strains and/or the same strain under varying conditions holds significant importance for propelling the advancement of nanotechnology and enhancing the versatility of nanomaterials in a wider range of applications.

There is a widespread consensus that selenium (Se) serves as a “natural antidote” for heavy metals, playing a crucial role in the health of animals and plants. Although Se was not considered an indispensable element for plant growth, it significantly improved plants’ ability to withstand stress, augmented their photosynthetic efficiency, and fortified their antioxidant defenses [10,14]. Provided in an optimal dosage, the positive impacts of Se ultimately contributed to ameliorating plant development and boosting agricultural crop productivity. Compared to another inorganic selenium, SeNP exhibited lower toxicity, higher biological activity, and ameliorated biological ability, thus rendering them advantageous in mitigating heavy metal stress in plants [15,16]. To counteract the toxicity of heavy metals, some studies had demonstrated that SeNPs could reduce the absorption of heavy metals and regulate the physiological metabolism of plants through forming insoluble compounds with heavy metals [5,9]. The biogenic SeNPs alleviated the detrimental effects antimony (Sb) in rice by restricting Sb absorption, bolstering antioxidant defense systems, and modulating the expression patterns of genes responsive to stress conditions [17]. By means of extensive physiological and transcriptomic analyses, Zeeshan et al. uncovered the mechanisms behind how SeNPs alleviate arsenite (AsIII) stress in soybean (*Glycine max* L. (Merr.)) roots through the modulation of antioxidant activities, metal transporter functions, and transcription factor expressions [18].

Endophytic fungi are capable of residing within the tissues of healthy plants without causing noticeable disease symptoms in their host plants [19]. These co-existing endophytic fungi were found to empower plants with resilience against heavy metal stress. Many studies have demonstrated that endophytic fungi not only stimulate plant growth by synthesizing hormones like IAA and ACC but also bolster plants’ tolerance to heavy metal stress by mitigating ROS, modulating heavy metal absorption and translocation, and minimizing metal accumulation within the plant body [20]. Over the past two decades, endophytic fungi have garnered significant interest from scientists due to their capability of aiding plants in coping with heavy metal toxicity. Additionally, some endophytic fungi have been found to possess the capability of synthesizing nanoparticles [21,22,23], which added another dimension to their potential value and applications.

This study screened an endophytic fungus KL19 capable of producing SeNPs and exhibiting Cd tolerance from among the 20 endophytic fungi both in *Kadsura angustifolia* and *Schisandra sphenanthera*. The objective was to investigate the individual effects of the endophytic fungus KL19 and its derived SeNPs on the morpho-physiological parameters, oxidative stress, and metal accumulation and translocation in small-leaf spinach plants grown in Cd-contaminated soil. Moreover, the taxonomic identification of strain KL19 and production optimization of biogenic SeNPs followed by their morpho-physical characterization were also performed in this study.

## 2. Results

### 2.1. Screening and Characterization of the Endophytic Fungus

#### 2.1.1. Screening of Effective Strains for Synthesizing SeNPs

**Figure 1:**
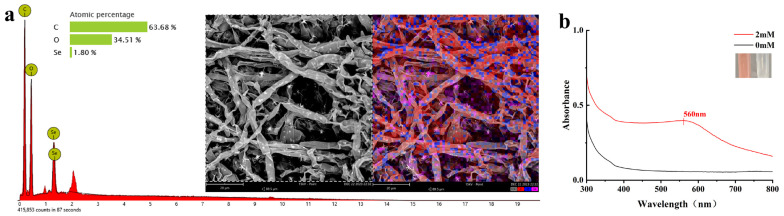
(**a**) The SEM-EDX image of the strain KL19 after being incubated in PDB containing 2 mM Na_2_SeO_3_ for 5 days. (**b**) Absorption spectrum of extracellular SeNPs biosynthesized by strain KL19 under 2 mM Na_2_SeO_3_.

After four days of cultivation on plates containing 2 mM Na_2_SeO_3_, 20 strains of endophytic fungi exhibited diverse growth patterns. Especially, strain KL19 exhibited superior growth performance compared to the other strains and underwent a noticeable transformation, developing a pronounced red–orange coloration in comparison to the control group (Figure S1). The outcomes pertaining to Cd and Na_2_SeO_3_ tolerance tests conducted in vitro showed that it exhibited a certain degree of tolerance (TI = 0.15 and 0.13, respectively) even when mycelium was exposed to 1000 mg/kg of Cd and 27 mM of Na_2_SeO_3_. Figure 1a presents an SEM-EDX image which revealed the presence of needle-like selenium crystals and round selenium particles on the surface of the hyphae and within the interstitial spaces between them. The reddish–orange supernatant showed an absorption peak around 560 nm in Figure 1b, which is a typical plasmon band for SeNPs [24]. These observations underscored the potential of strain KL19 to synthesize SeNPs. As a result, strain KL19 was chosen as the biological material for the synthesis of SeNPs in this experimental investigation. For convenience, all subsequent acquisitions of SeNPs would exclusively be sourced from the extracellular solution.

#### 2.1.2. Morphological and Molecular Identification

**Figure 2:**
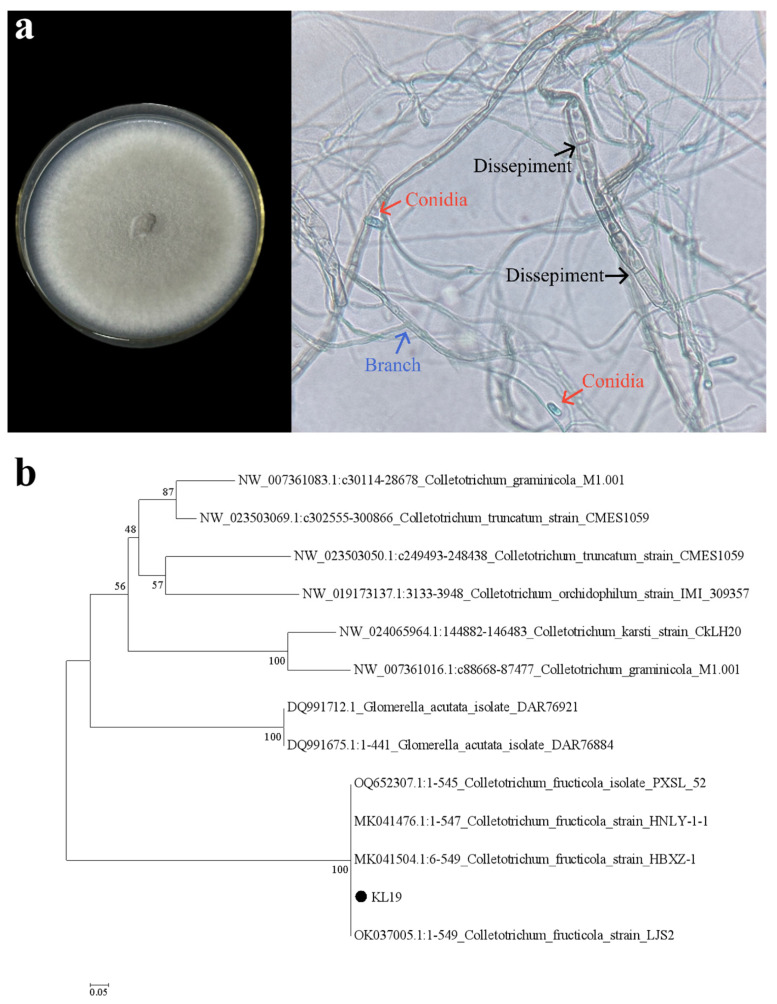
(**a**) The morphological characteristics of strain KL19. (**b**) Phylogenetic tree based on ITS sequences.

The morphological characteristics of KL19 are presented in Figure 2a. Its aerial mycelium exhibited a range of colors from white to pale, appearing dense and cotton-like, with a grayish surface grading from light to dark toward the center. Microscopically, the hyphae revealed smooth cell walls, septate, and branched. Conidia, hairless and unisporous, were formed directly on the hyphae, transparent and smooth, lacking septa, cylindrical with rounded or slightly pointed ends (Figure 2a). The morphological characteristics were consistent with those described in a previous study [25]. Additionally, according to the phylogenetic analysis presented in Figure 2b, strain KL19 clusters togethered with the reference strain of *C. fructicola*, exhibiting a high homology rate of 100%. Combined with its morphological characteristics, strain KL19 was identified as *C. fructicola* (Accession # PP930589).

### 2.2. Fitting Models of SeNPs Yields and Analysis of the BBD

Utilizing the BBD, the various method variables and their levels had been systematically compiled and presented in Table 1. Subsequently, the experiments were performed to generate a model encompassing a total of 17 trials (Table S2). The data compiled in Tables S2 and S3 offered experimental outcomes and variance analysis (ANOVA) pertaining to the BBD, specifically regarding Se^0^ content. Through model analysis, the correlation between the experimental results and the independent variables was elucidated using the following equation.

Y = 0.8497 + 0.2298A + 0.0561B − 0.0685C − 0.1764AB + 0.0454AC + 0.0215BC

The 2FI equation could serve as a tool for forecasting the response of each parameter at a specific level. As shown in Table S3, the established regression model exhibited statistical significance at *p* < 0.0001 and a coefficient of variance of 7.83%, while the minor discrepancy between R^2^ (0.9334) and adjusted R^2^ (0.8934) confirmed its statistical reliability [26]. The model’s accuracy and reliability were validated by its substantial F-value of 23.36, which was accompanied by a probability level of *p* < 0.0001. Furthermore, the ‘lack of fit F-value’ of 3.82 indicated that the lack of fit was negligible when contrasted with the pure error.

#### 2.2.1. Analysis of 2D and 3D Response Surface Plots

**Figure 3:**
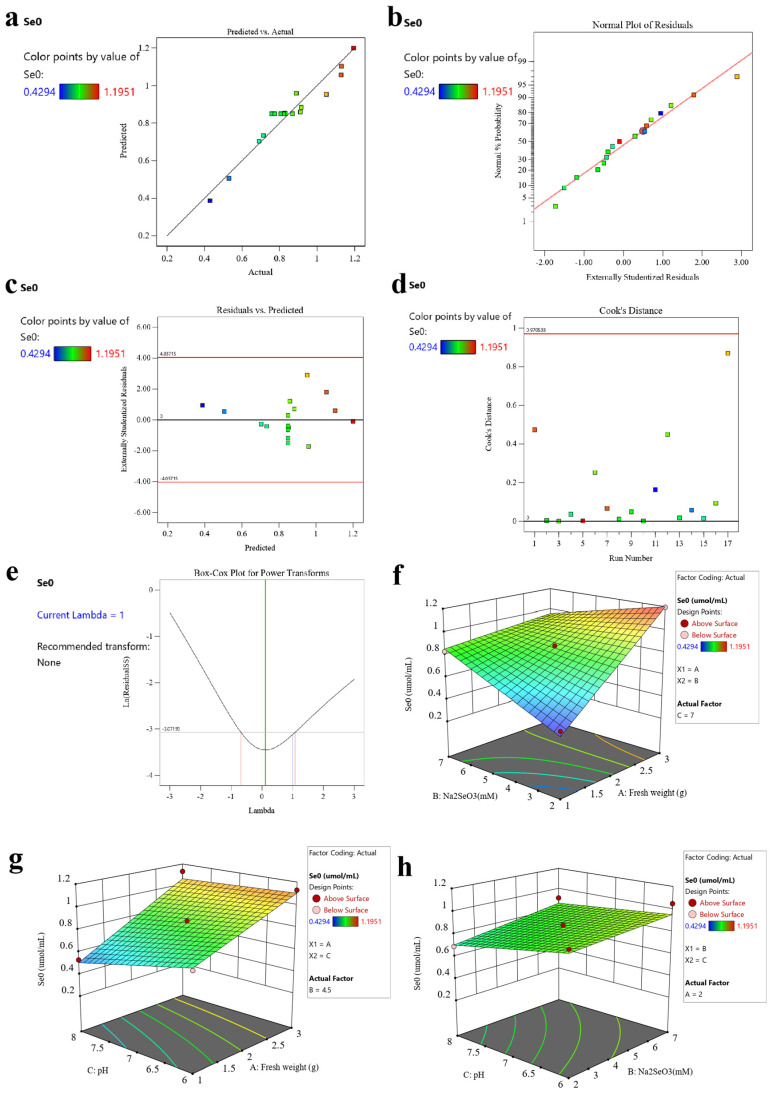
(**a**) Visualization of predicted and actual measurements. (**b**) Normal probability plot. (**c**) Visualization illustrating the relationship between residuals and predictions. (**d**) Visualization of Cook’s distance plotted versus run number. (**e**) Visualization of Box–Cox transformations for power scaling. (**f**) The interactive effects between the initial concentration of Na_2_SeO_3_ and the biomass of strain KL19 on the yields of SeNPs. (**g**) The interactive effects between the pH and the biomass of strain KL19 on the yields of SeNPs. (**h**) The interactive effects between the pH and the initial concentration of Na_2_SeO_3_ on the yields of SeNPs.

Figure 3a displayed a plot comparing the actual and predicted yields of SeNPs, where it was evident that the experimental data points were relatively evenly distributed on both sides of the fitted curve. Figure 3b demonstrated the absence of abnormal residuals, while Figure 3c depicted a random distribution of residuals around the predicted values, which were both indicating a high degree of predictability inherent in the design. Figure 3d,e revealed that the standard deviation was less than 1 and fell within the range of 0 to 1. All these results clearly demonstrated the optimal fitting effect of the mode [27].

It was evident from Figure 3f that the production of SeNPs positively correlated with the increase in Na_2_SeO_3_ concentration and fungal biomass. Especially, a more pronounced interaction was observed between biomass and Na_2_SeO_3_ concentration when the biomass was maintained at lower or moderate levels. From Figure 3g,h, the quantity of SeNPs rose as the pH decreased even the interactions between pH and selenite concentration, and the pH and fungal biomass are relatively insignificant. Additionally, Table S3 unequivocally reveals that the primary and secondary relationships of the interaction among these three variable factors in the production of SeNPs were AB > AC > BC.

#### 2.2.2. Optimization and Validation

After optimization using the RSM, the determined parameters for the synthesis of SeNPs were outlined as follows: fungal biomass of 2.62 g, Na_2_SeO_3_ concentration of 4.56 mM, and pH of 6.25. Under these conditions, the theoretical production of SeNPs was 1.02 mM. To verify the reliability of the response model, a conditional experiment with the optimal process parameters was designed. Following three replicated tests, the actual production amounted to 1.06 mM (as detailed in Table 2), closely aligning with the predicted data. This conclusively demonstrated the effectiveness of the model.

### 2.3. Biogenic SeNPs Characterization

**Figure 4:**
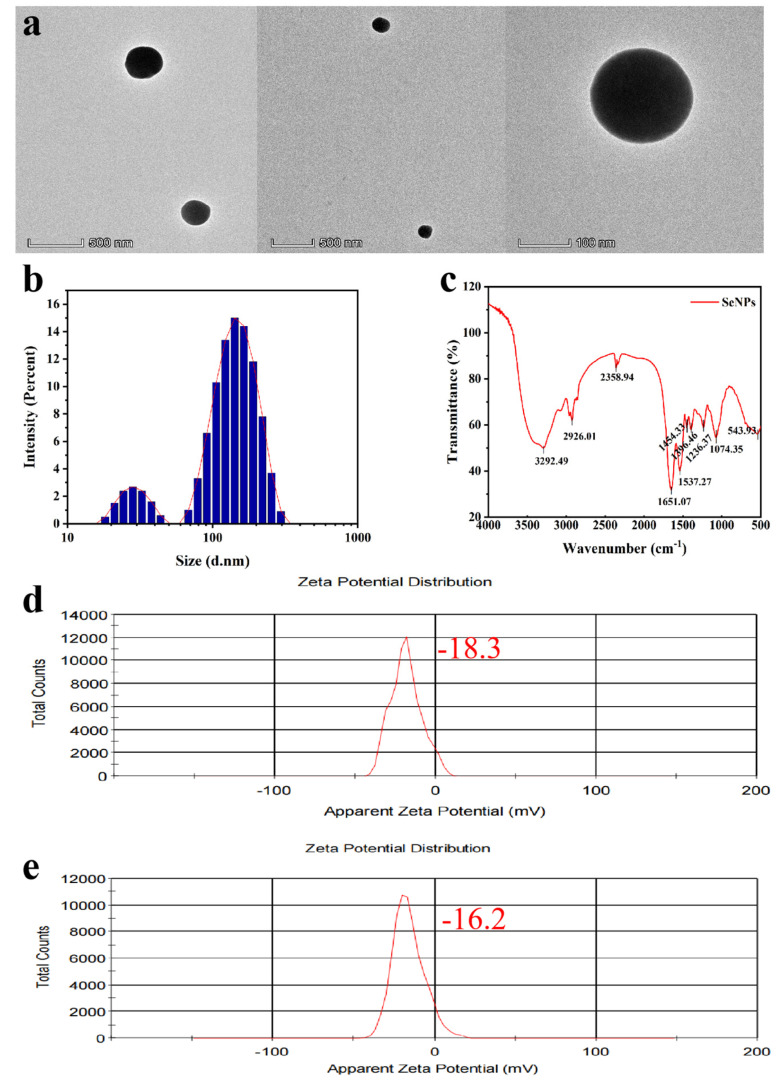
(**a**) TEM image depicting extracellular nanoparticles. (**b**–**e**) SeNPs size distribution and zeta potential analysis. (**c**) FTIR spectroscopy of SeNPs.

The TEM images in Figure 4a displayed that the clear-outlined SeNPs uniformly dispersed without obvious aggregation and possessed a typical spherical structure with a size under 200 nm, which corresponded to the result of DLS shown in Figure 4d,e.

The intensity distribution depicted in Figure 4b represents the percentage of intensity as a function of the particle diameter. Based on this histogram, it has been observed that SeNPs took on a Z-average diameter of 144.8 nanometers, accompanied by a polydispersity index of 0.316, indicative of a uniform size distribution, and a zeta potential of −18.3 millivolts (Figure 4d). After a period of three months, the zeta potential slightly shifted to −16.2 millivolts (Figure 4e), suggesting that the distribution consisted of a monomeric mode without aggregates and exhibited good dispersibility.

FTIR spectroscopy (Figure 4c) was performed to identify functional groups responsible for the stabilization and capping of SeNPs, including hydroxyls, saturated C-H, carbonyls, aromatic rings, methyls, and C-O. While peaks at 3292.49 cm^−1^, 1651.07 cm^−1^, and 1537.27 cm^−1^ suggested hydroxyls, carbonyls, and aromatic structures, respectively, confirmation required additional analysis. Peaks at 2926.01 cm^−1^, 1454.33 cm^−1^, 1396.46 cm^−1^, and 1074.35 cm^−1^ indicated the presence of saturated C-H, methyls, and C-O bonds [28]. The 543.93 cm^−1^ peak’s attribution was complex and required further study. Given the complexity of spectral interpretation and potential spectral overlap, definitive structural confirmation necessitated the integration of other analytical techniques (e.g., nuclear magnetic resonance, mass spectrometry) and experimental evidence.

### 2.4. Soil-Pot Experimentation

#### 2.4.1. Strain KL19 and SeNPs Improved the Growth of Small-Leaf Spinach under 

##### Cd Stress

**Figure 5:**
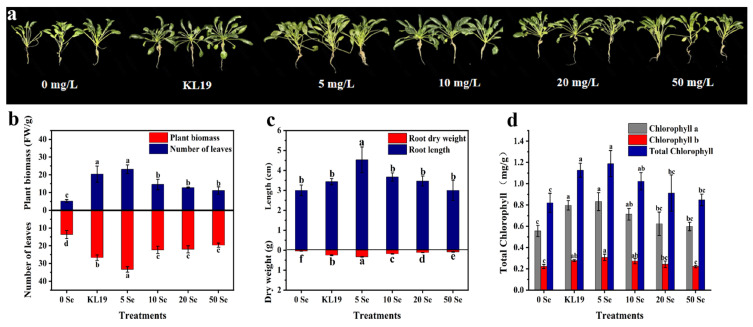
Effects of strain KL19 and its derived SeNPs at different concentrations on growth parameters and pigments of small-leaf spinach under Cd stress. (**a**) Representative images of small-leaf spinach plants. (**b**) Biomass and number of leaves. (**c**) Root dry weight and root length. (**d**) Chlorophyll a,b and total chlorophyll contents. Different letters above the bars signify statistically significant differences at a *p*-value of < 0.05, as determined by one-way ANOVA followed by the LSD test.

It was discovered that inoculating strain KL19 and introducing SeNPs (5–50 mg/L) into artificially Cd-contaminated soil could effectively enhance the growth of small-leaf spinach seedlings (Figure 5). Especially, seedlings treated with strain KL19 and 5 mg/L SeNPs were healthier and more robust, growing better than the control and other treatment groups. In comparison to the control, the root length of seedlings subjected to strain KL19 exhibited a substantial 0.92-fold increase in leaf count, a remarkable 2.94-fold enhancement in fresh weight, and a significant 4-fold surge in root dry weight, although they showed a minor increase of just 0.14-fold in root length. Furthermore, plants treated with 5 mg/L SeNPs displayed even more profound improvements with a substantial 1.36-fold leap in leaf count, a substantial 3.46-fold augmentation in fresh weight, a marked 5.6-fold elevation in root dry weight, and a noteworthy 0.51-fold extension in root length (Figure 5b,c).

In this study, strain KL19 and SeNPs also exhibited an improvement in chlorophyll levels in comparison to the control group (Figure 5d). However, as the SeNPs concentration increased, the positive impact on chlorophyll levels gradually diminished. Statistically significant increases in chlorophyll levels were observed in seedlings treated with strain KL19 and 5 mg/L SeNPs compared to the control. In the strain KL19-treated group, chlorophyll a increased by 0.43-fold, chlorophyll b increased by 0.27-fold, and total chlorophyll increased by 0.38-fold. In addition, exposure to 5 mg/L of selenium nanoparticles (SeNPs) resulted in significant elevations of 0.48-fold in chlorophyll a, 0.41-fold in chlorophyll b, and 0.45-fold in total chlorophyll content compared to the untreated control group. These findings demonstrated that the application of stain KL19 and 5 mg/L SeNPs exerted a profound influence in mitigating the adverse impacts of Cd toxicity on spinach plants, especially with 5 mg/L SeNPs.

#### 2.4.2. Effect of Strain KL19 and SeNPs on Spinach Leaf Physiology

**Figure 6:**
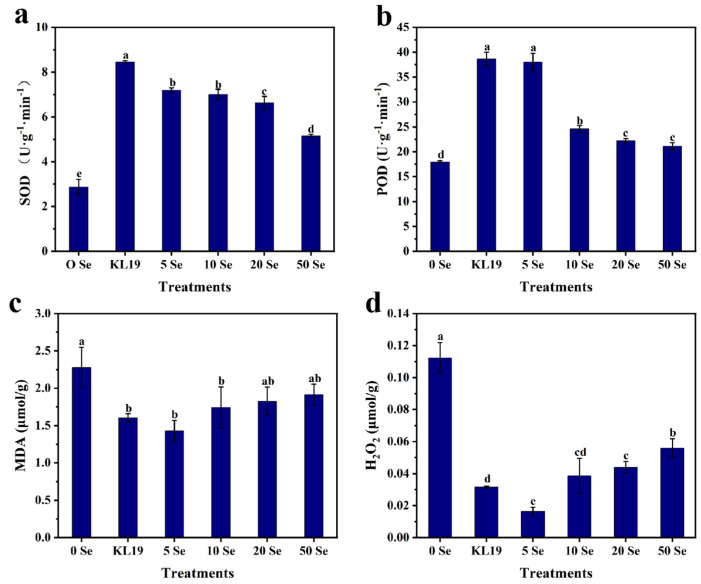
Effects of strain KL19 and SeNPs at different concentrations on SOD (**a**), POD (**b**), MDA (**c**), H_2_O_2_ (**d**) in Cd-stressed small-leaf spinach. Different letters above the bars signify statistically significant differences at a *p*-value of < 0.05, as determined by one-way ANOVA followed by the LSD test.

To assess the impact of strain KL19 and various SeNPs concentrations on small-leaf spinach’s endogenous antioxidant system, their SOD, POD, MDA, and H_2_O_2_ levels were analyzed. Both strain KL19 and SeNPs enhanced small-leaf spinach’s antioxidant capacity compared to the control group with strain KL19 and 5 mg/L SeNPs exhibiting the most significant and similar effects. After administering strain KL19, SOD levels surged 1.95-fold and POD levels rose 1.15-fold, while MDA and H_2_O_2_ concentrations decreased by 0.30-fold and 0.71-fold, respectively. With the introduction of 5 mg/L SeNPs, SOD levels jumped 1.51-fold and POD levels increased 1.11-fold, whereas MDA and H_2_O_2_ levels plummeted by 0.37-fold and 0.82-fold, respectively (Figure 6). Therefore, both strain KL19 and 5 mg/L SeNPs boosted antioxidant capabilities significantly, achieving this by lessening oxidative damage and boosting antioxidant enzymes and/or related substances. Notably, 5 mg/L SeNPs displayed a more potent effect in minimizing oxidative harm.

#### 2.4.3. Cd Uptake and Translocation and Se Influx in Small-Leaf Spinach

**Figure 7:**
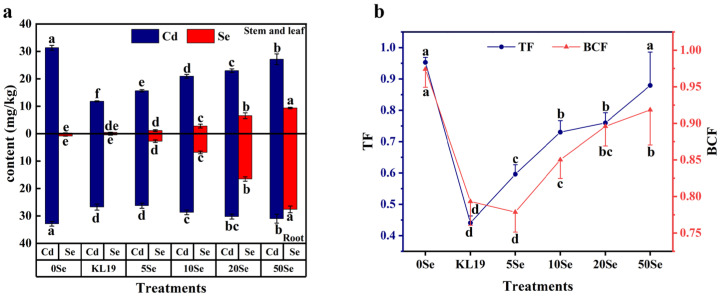
(**a**) The contents of Cd and Se in the aboveground and underground parts of Cd-stressed small-leaf spinach under the treatment of strain KL19 and SeNPs at different concentrations. (**b**) The TF and BCF of Cd in Cd-stressed small-leaf spinach under the treatment of strain KL19 and SeNPs at different concentrations. Different letters above the bars and lines signify statistically significant differences at a *p*-value of < 0.05, as determined by one-way ANOVA followed by the LSD test.

Small-leaf spinach exhibited a remarkable ability to enrich SeNPs synthesized by strain KL19, and the Se concentration in the plants positively correlated with the injected SeNPs concentration (Figure 7a). Different concentrations of SeNPs had various impacts on the Cd concentration in plants. As depicted in Figure 7a, the Cd concentration consistently exceeded that of the aboveground part in the underground section. In contrast to the untreated control group, the application of 5 mg/L SeNPs exhibited the most significant reduction in Cd content with decreases of 0.50-fold in the aboveground parts and 0.20-fold in the underground parts, respectively. Apart from the 5 mg/L SeNPs treatment group, the strain KL19 also demonstrated promising results, reducing the concentration of Cd in the aboveground and underground parts of the plants by 0.62-fold and 0.19-fold, respectively.

In addition, the TF and BCF of Cd under different concentrations of SeNPs and strain KL19 are presented in Figure 7b. In contrast to the untreated control group, the TF and BCF decreased most significantly in the groups treated with 5 mg/L SeNPs and strain KL19, with TF decreasing by 0.37-fold and 0.54-fold, and BCF decreasing by 0.20-fold and 0.18-fold, respectively. Additionally, high concentrations of 50 mg/L SeNPs reduced the TF and BCF of Cd in small-leaf spinach, but not significantly, with TF and BCF decreasing by 0.08-fold and 0.06-fold, respectively. These results indicated that both strain KL19 and SeNPs at different concentrations hindered the absorption of Cd by small-leaf spinach and its transportation within the plants. It was noteworthy that compared to the provision of SeNPs, the provision of strain KL19 led to a notable reduction in Cd accumulation in aboveground parts under Cd stress, indicating that strain KL19 may adsorb a certain amount of Cd in the soil, mitigating its entry into the plants.

## 3. Discussion

The employment of selenium in various forms within agricultural practices has been recognized as a potent strategy to mitigate the toxicity of heavy metals such as lead (Pb), cadmium (Cd), mercury (Hg), and chromium (Cr) in vegetable crops. This is achieved by bolstering the antioxidant defense mechanisms within the plants, ultimately minimizing or averting detrimental effects on plant tissues [15,29]. In comparison to elemental selenium, SeNPs synthesized by microorganisms exhibited superior properties, particularly in terms of their environmentally friendly and biocompatible nature [30]. The current study employed a Cd-resistant endophytic *C. fructicola* strain KL19 to produce SeNPs. The molecular rank of the strain was confirmed as *C. fructicola* after the analysis of the ITS gene sequence. In the literature, *Colletotrichum* sp. has been reported to be capable of extracellularly synthesizing SNPs and AlNPs [31]. In 2022, Mukherjee et al. identified two endophytic *Colletotrichum* strains, ALE15 and ALE18, with high Cd tolerance (up to 1000 and 750 µg/mL) that accumulated Cd in their cells as a survival strategy under Cd stress [32]. Additionally, Wang et al. reported that three distinct strains, designated as F1 (*Mucor circinelloides*), F2 (*Curvularia lunata*), and F3 (*Clonostachys rosea*), exhibiting remarkably high resilience to Cd and an ability to immobilize it, were successfully isolated from Cd-tolerant soybean varieties, the rhizosphere environment surrounding the plant roots, as well as the main soil matrix. These strains possessed the capacity to significantly diminish the solubility of Cd in water and its extractability using EDTA (ethylenediaminetetraacetic acid) within the rhizosphere soil, potentially mitigating Cd’s bioavailability and toxicity [33]. This study marked the first discovery of the *C. fructicola*’s remarkable tolerance to Cd and its capacity to synthesize SeNPs intracellularly and then release them extracellularly, paving the way for their innovative application in mitigating Cd stress in small-leaf spinach seedlings.

The extracellular conversion of selenite into elemental selenium by microorganisms has been acknowledged as an effective detoxification mechanism [7]. The formation of SeNPs might be linked to the chemical and enzymatic reduction pathways, which is a process that occurred within the confines of the cytoplasm. Following this reduction, the SeNPs were likely expelled from the cells through a mechanism analogous to cell lysis [34]. Observed through SEM-EDX imaging (Figure 1a), needle-shaped selenium crystals and rounded selenium particles were found adhered to the surface of the mycelium as well as within the interstices between the hyphae. Kumar et al. revealed that selenium spheres which were agglomerations of nanoscale subunits crystallized in a hexagonal lattice could be transformed into selenium wires and rods by modifying the pH and composition of the precipitated dispersions, which was followed by incubation at moderate temperatures for an extended period [35]. Therefore, it was suspected that the needle-shaped formations observed in this study were the outcome of recrystallization of the initial selenium spheres. In addition, SeNPs colloidal solution could induce a strong absorption of ultraviolet and visible light wavelengths and showed red due to the surface plasmon resonance (SPR) of SeNPs. It was observed from UV-Vis absorption spectroscopy imaging (Figure 1b) that SeNPs existed in the extracellular solution with the maximum absorption peak at 560 nm (Figure 1a,b). Some literature sources have elucidated the potential mechanisms governing the release of intracellular NPs into the extracellular environment, primarily encompassing direct secretion, vesicular excretion, and cell lysis [36,37]. In this study, after five days of culturing strain KL19 in a solution of Na_2_SeO_3_, notable color changes and the emergence of distinct absorption peaks were finally observed in the extracellular environment. This phenomenon has been hypothesized to stem from the release of biosynthesized SeNPs into the extracellular space by strain KL19, potentially occurring through cell lysis following prolonged cultivation periods. Despite relying solely on SEM-EDX and UV-visible absorption spectroscopy, the current study has been limited in its observations, solely confirming the presence of SeNPs on and between the hyphae without conclusive evidence to verify their cytoplasmic origin. Consequently, the precise location and mechanism of SeNPs formation remain elusive and warrant further exploration and research.

The physiological characteristics of microbial strains, including morphology, growth rate, and resistance, significantly impacted the synthesis of nanoparticles. To optimize their production, it was crucial to cultivate them under suitable conditions, such as temperature, ion concentration, and pH. By fine tuning these factors, the yields and quality of NPs were enhanced. RSM, specifically the BBD, serves as an efficient and economical means for assessing the impacts of diverse factors [38]. Currently, numerous reports have documented the application of BBD in optimizing the production of NPs [38,39]. Using BBD, the strain KL19 was found to produce the highest amount of SeNPs under conditions of biomass of 2.62 g, initial Na_2_SeO_3_ of 4.56 mM, and a pH of 6.25. The biomass level directly correlated with the number of microorganisms involved in nanoparticle synthesis. A higher biomass meant more cells participating in ion reduction and NPs formation, leading to increased production. However, excessive biomass may result in intracellular particle accumulation, limiting extracellular release. Additionally, the tolerance tests revealed that strain KL19 exhibited optimal reduction and SeNPs release capabilities at a Na_2_SeO_3_ concentration of 4.56 mM, which was likely due to its detoxifying abilities. As expected, the optimal pH for SeNPs production aligned with previous reports on the growth conditions of related microorganisms [40], further supporting the role of strain KL19 in efficient SeNPs production.

The locations of the maximum peaks in the UV-Vis absorption spectrum varied depending on the colloidal sizes [41]. Utilizing DLS analysis, the average size of SeNPs was 144.8 nanometers, corresponding precisely with the location of the peak maximum observed in its UV-Vis spectrum. It was well known that the magnitude of zeta potential served as an indicator of the strength of electrostatic repulsion among charged particles within a dispersion system. Higher zeta potential values signified greater stability, enabling solutions or dispersions to effectively resist aggregation [42]. Interestingly, many factors such as pH value and particle concentration affected zeta potential [43]. In order to evaluate the durability or consistency of the SeNPs in this investigation, a thorough analysis was undertaken examining the zeta potential of the colloidal solution stored at 4 °C. The result revealed that the zeta potential exhibited minimal variation over time, with a value of −18.3 on the first day and −16.2 after three months (Figure 4d,e), both displaying narrow peaks, underscoring the excellent stability and monodispersity of SeNPs prepared under optimal conditions. As depicted in Figure 4a, TEM confirmation corroborated that the SeNPs exhibited a rounded morphology and displayed remarkable dispersibility. Furthermore, analysis via FTIR indicated that the surface of SeNPs derived from strain KL19 was adorned with different functional groups, revealing a protein coating. Similarly, the surfaces of biologically sourced SeNPs from *Trichoderma* sp. WL-Go were coated with proteins, which imparted extracellular stability and biocompatibility to these SeNPs and/or acted as reducing agents and capping agents [24].

Spinach exhibited a remarkable ability to accumulate a large amount of Cd, and as the Cd dosage increased, the dry weight of the plant’s leaves and roots suffered negative impacts [44]. The pot experiments showed that strain KL19 and its derived SeNPs had effectively lessened the uptake and translocation of Cd in plants, as well as modulated the activities of antioxidant enzymes to mitigate oxidative stress, ultimately alleviating the detrimental impacts of Cd stress on spinach plants.

The most evident signs of Cd toxicity included reduced biomass and photosynthesis [14]. Cd distributed evenly within the chloroplasts and mitochondria of *Brassica napus* L., causing lipid oxidation and structural distortions in thylakoid membranes [45]. Similarly, Cd stress led to changes in the ultrastructure of the four *Dendranthema morifolium* cultivars commonly used as ornamental plants, including alterations in chloroplasts, grana, lamellae, thylakoids, and stroma, significantly inhibiting the total chlorophyll content, as well as the contents of chlorophyll a, chlorophyll b, and carotenoids [46]. Oxidative stress disrupted thylakoid levels, accelerating the degradation of chlorophyll in thylakoids by chloroplast enzymes located on the inner membrane of chloroplasts. Generally, the addition of Se could promote plant carbohydrate metabolism and accelerate chlorophyll biosynthesis [47]. In 2021, Qi et al. found that the beneficial concentration range of SeNPs for *Brassica napus* L. was broader than that of SeO_3_^2−^ and had a more significant effect on enhancing root cell viability under Cd stress [45]. Gu et al. suggested that SeNPs accelerated the synthesis of chlorophyll and cellular growth by enhancing the carbon fixation rate in green algae [48]. Among these tested concentrations, 5 mg/L SeNPs showed the most beneficial effect, significantly enhancing the fresh weight (3.46-fold), leaf count (1.36-fold), root length (0.51-fold), root dry weight (5.6-fold), and total chlorophyll content (0.45-fold) in Cd-stressed spinach plants (Figure 5). However, it was noteworthy that high concentrations of selenium can exert toxic effects on plants [49]. As evident from Figure 5d, despite the increase in SeNPs concentrations, chlorophyll contents suffered a detrimental impact with no significant differences observed between the 20 and 50 mg/L SeNPs treatment groups compared to the control. In Li et al.’s study, excessively high concentrations of SeNPs led to severe damage to the root tip cells of pepper under Cd stress [16]. Additionally, nitrate reductase played a pivotal role in facilitating nitrate reduction and nitrogen assimilation, providing essential nitrogen sources and nutritional support for chlorophyll synthesis. Consistent with this, Sara et al. discovered that low doses of SeNPs enhanced biomass accumulation in bittermelon (*Momordica charantia*) and augmented the activity of leaf nitrate reductase, whereas concentrations of 10 mg/L and above exhibited pronounced toxicity [50]. In other words, the application of SeNPs at optimal concentrations led to an improved growth of small-leaf spinach under abiotic stress, suggesting that these concentrations might also stimulate the synthesis of carbohydrates in small-leaf spinach plants.

Since Cd was not easily translocated in the phloem, most plants accumulate more Cd in their roots than in their stems [51]. In this paper, there was a higher Cd content in the roots of small-leaf spinach compared to its stem and leaves, but the difference was not substantial, which was likely due to the duration of exposure to Cd-contaminated soil and soil conditions [52]. Totally, the accumulation of Cd within root structures and cell walls constituted a vital detoxification mechanism, serving as a critical barrier that effectively hindered the penetration of Cd into the internal plant tissues and cellular compartments, including the protoplasts [53]. As is commonly known, the accumulation of Cd within cells depended on the membrane permeability and the activity of cation channels. Filek et al. showed that Se altered the fatty acid saturation in both the aerial and underground parts of oilseed rape, affecting the permeability of the plasma membrane and reducing Cd absorption, all without inflicting any detrimental effects on plant growth or health [54]. Li et al. exhibited that SeNPs successfully mitigated the accumulation of Cd in peppers by modulating the metabolic route responsible for lignin biosynthesis and influencing hormone-signaling cascades [16]. Moreover, SeNPs possessed the capacity to swiftly transform SeMet in the root system, forming complexes with Cd, effectively sequestering it and preventing its translocation to the upper sections of the peppers [55]. Here, despite not knowing the specific reason, all treatments with SeNPs at different concentrations resulted in varying degrees of decrease in the translocation factor and concentration factor of Cd in spinach (Figure 7).

The primary culprit behind compromised plant growth was the oxidative stress brought on by Cd [56], and the intracellular clearance of toxic ROS was primarily achieved through the concerted action of enzymatic and non-enzymatic antioxidants. SeNPs could reduce oxidative damage induced by pesticides in tea plants by decreasing the levels of H_2_O_2_, O^2−^, and MDA [57]. SeNPs have been reported to elevate the levels of Ca^2+^-associated transcription factors and enzymes in plants experiencing calcium (Ca) imbalance under Cd stress, indicating their capability to regulate Ca^2+^ flow and cellular reactions for ROS balance, ultimately aiding plants in heavy metal detoxification [45]. In 2023, Zeeshan et al. showed that the introduction of SeNPs led to an enhanced regulation of glutathione peroxidase (GmGPX) and ascorbic acid gene expression, which facilitated the metabolism of H_2_O_2_, mitigating the detrimental effects on soybean roots caused by arsenic (As) stress [18]. In the same year, Gu et al. indicated that the addition of SeNPs significantly upregulated genes such as SODA (Unigene0008135) and SODB, inducing the accumulation of antioxidant enzymes [48]. Similarly, the current study revealed that the application of SeNPs in small-leaf spinach enhanced the accumulation of antioxidant enzymes SOD and POD, leading to reduced levels of MDA and H_2_O_2_ (Figure 6).

It was found that stain KL19 had an inhibitory impact on Cd accumulation in small-leaf spinach. The biomass and chlorophyll contents of the small-leaf spinach in the KL19 treatment group ranked second, only next to the 5 mg/L SeNPs treatment. The SOD and POD contents of strain KL19-treated small-leaf spinach both exceeded those of the 5 mg/L SeNPs treatment, but the MDA and H_2_O_2_ levels in the 5 mg/L SeNPs treatment group exhibited a reduction compared to the strain KL19 treatment group. Therefore, it could be speculated that strain KL19 primarily bound with Cd in the soil, forming heavy metal complexes that resisted absorption by plant roots while having some adverse effects on samll-leaf spinach, which was consistent with the view [58]. This binding process effectively prevented Cd from being absorbed and accumulated in the small-leaf spinach, significantly enhancing their biomass under Cd stress conditions. More importantly, endophytic fungi represent an incredibly rich source of bioactive metabolites which not only provide endophytic fungi with survival advantages but also play a pivotal role in enhancing the stress tolerance of their host plants [59]. In Aziz et al.’s research, the endophytic fungus *Aspergillus violaceofuscus* Ch06 from Chilli produced heightened levels of IAA and other metabolites like sugars, proteins, phenolics, and flavonoids under Cd and Cr heavy metal stress, significantly boosting the growth of okra plants exposed to these heavy metals [60]. Undoubtedly, more profound research on the impact of *C. fructicola* on the growth of small-leaf spinach needs to be conducted. Additionally, given *C. fructicola’*s role as a “producer” of SeNPs, it may possess the capacity to reduce and release less toxic SeNPs within spinach plants or their rhizosphere under selenate conditions. The synergistic interaction between *C. fructicola* and the produced SeNPs may potentially mitigate the adverse effects of Cd stress on spinach through various mechanisms, including enhancing selenium bioavailability, stimulating plant antioxidant systems, diminishing Cd uptake and accumulation, and improving soil quality. Future research endeavors should further explore the intricate mechanisms and efficacy of this synergistic interaction, providing a solid theoretical foundation and practical technical support for its application in agricultural practices. In the current investigation, the influence of 5 mg/L SeNPs in alleviating the detrimental consequences imposed by Cd stress on small-leaf spinach was profoundly remarkable. The reason might be that SeNPs actuated a robust accumulation of antioxidant enzymes, leading to a substantial reduction in MDA and H_2_O_2_ levels that were otherwise induced by Cd stress in the spinach plants. This mechanism further accelerated the excretion of Cd ions and significantly bolstered the cellular resilience against Cd toxicity, leading to a perceptible improvement in the chlorophyll content responsible for photosynthesis and an overall augmentation in the biomass of the small-leaf spinach.

## 4. Materials and Methods

### 4.1. Experimental Reagents

Sodium selenite (Na_2_SeO_3_, purity ≥ 99%), sourced from Chongqing Yuexiang Chemical Co., Ltd. (Chongqing, China), was acquired. A concentrated stock solution of 1 molarity was prepared by dissolving Na_2_SeO_3_ in deionized water and subsequently sterilized through filtration to ensure purity. All additional analytical-grade chemicals and reagents were procured from reputable suppliers, including Chongqing Titanium New Chemical Co., Ltd. and Yuexiang Chemical Co., Ltd., both situated in Chongqing, China.

### 4.2. Screening of Endophytic Fungi

The aim of this experiment was to screen for a specific strain among the 20 endophytic fungi both in *Kadsura angustifolia* and *Schisandra sphenanthera* (Table S1) [61], which possesses dual capabilities of both tolerating and reducing Na_2_SeO_3_ into SeNPs while also exhibiting robust tolerance towards Cd.

By inoculating 20 endogenous fungal strains onto potato dextrose agar (PDA) plates containing 2 mM Na_2_SeO_3_, the growth and color changes of the strains under constant temperature of 28 °C were observed to preliminarily assess their ability to produce selenite-reducing agents. Following the method established by Valix and Loon [62], the best-performing strain was selected for further growth experiments to determine its tolerance to Na_2_SeO_3_ and CdCl_2_·2.5H_2_O.

To further confirm whether the selected strain possesses the ability to extracellularly secrete SeNPs, the fungal strain was initially propagated in a medium composed of potato dextrose broth (PDB) for the purpose of accumulating a substantial quantity of biomass. Subsequently, it was suspended in sterile water containing 2 mM Na_2_SeO_3_ and allowed to grow under controlled conditions of 150 revolutions per minute (rpm) and a temperature of 28 degrees Celsius (°C) for a period of 5 days. Following this, the fungal surface characteristics and element changes observed were visualized utilizing a scanning electron microscope (SEM) integrated with an energy-dispersive X-ray spectroscopy (EDS) apparatus (Phenom Pro, Phenom-World, Eindhoven, The Netherlands) [63]. Finally, 180 μL of the filtrate was pipetted and scanned using an enzyme-labeled instrument (Bio-Rad, Hercules, CA, USA) between 300 and 700 nm with the wavelengths recorded.

#### Taxonomic Identification and Phylogenetic Analysis of Strain KL19

The strain KL19, capable of synthesizing SeNPs, was inoculated into PDA and incubated in a 28 °C constant temperature incubator. Using the cotton blue staining method [64], morphological characteristics such as hyphae and spores were observed under a compound light microscope at various magnifications, enabling morphological identification of the strain KL19.

For taxonomic identification, the DNA of the chilled mycelia in liquid nitrogen was extracted using the CTAB method [65]. The amplification of the fungal ITS fragments (ITS1-5.8S-ITS2 rDNA) was carried out using the universal primers ITS1 and ITS4 reported by White et al. [66], which was followed by PCR amplification and sequencing. The obtained sequences were subsequently uploaded to the NCBI database (https://www.ncbi.nlm.nih.gov/) for homology analysis and comparison with sequences in GenBank using BLAST search. After alignment, the sequences with the highest similarity and those belonging to the closest taxonomic groups were selected. MEGA5.1 software was utilized to perform sequence analysis on the ITS sequences of strain KL19 along with 10 other registered strains from the NCBI database. A phylogenetic dendrogram was generated employing the Neighbor-Joining (N-J) algorithm, incorporating 1000 resampling iterations for bootstrap analysis to enhance the robustness and reliability of the inferred evolutionary relationships [66], enabling the classification and identification of the strain KL19 at the species level.

### 4.3. Optimization and Acquisition of Extracellular SeNPs

Since extracellular SeNPs were readily accessible and can be purified efficiently, all subsequent experiments in this study utilized SeNPs secreted outside the cells. To ensure the vitality of the endophytic fungi, we followed the mycelial biomass method to cultivate the strain in PDB at 28 ± 1 °C and 150 rpm to determine its growth curve, harvesting the biomass at the stable period. Additionally, the BBD method was utilized within Design-Expert 13 software to optimize three factors: fungal biomass (wet weight 1–3 g), initial Na_2_SeO_3_ concentration (2–7 mM), and pH value (6–8) for maximizing SeNPs yields. All optimization experiments were conducted in a system containing 20 mL of Na_2_SeO_3_ solution, which was maintained at 28 ± 1 °C and 150 rpm. After five days of reaction, to measure extracellular SeNPs, we used double-layer filter paper (0.45 μm) to filter and collect the filtrate containing SeNPs. This filtrate was then subjected to ultrasonic disruption (100 W, 10 min) and centrifugation (5000 rpm, 5 min, 4 °C) to remove cellular debris. The supernatant containing SeNPs underwent additional centrifugation at 12,000 rpm for 30 min at 4 °C, resulting in the sedimentation of SeNPs. Subsequently, the precipitate was subjected to three rounds of rinsing with 1 M NaCl solution to effectively remove any lingering Na_2_SeO_3_ impurities. Finally, the concentration of biologically synthesized SeNPs was measured utilizing the protocol detailed by Biswas et al. [67].

### 4.4. Characterization of SeNPs

To ascertain the morphology and dimensions of bio-sourced SeNPs, a drop of the purified SeNPs solution under optimized conditions was applied onto a copper mesh grid. Following evaporation of the solvent, the grid was examined utilizing a transmission electron microscope (TEM) (Talos F200X, FEI, Czech Republic). The particle size and stability were further analyzed using particle size analysis and zeta potential measurement (DLS) (ZEV360011000, Malvern, UK). After being dried by a freeze dryer, the SeNPs powder was thoroughly mixed with potassium bromide (AR) in a ratio of 1:100. The mixture was then prepared for Fourier transform infrared scanning (FTIR) (IRPrestige-21, Shimatsu, Kyoto, Japan) within the 400–4000 cm^−1^ wavenumber range to investigate its interfacial properties.

### 4.5. Pot Experiment

#### 4.5.1. Soil Treatment and Planting of Small-Leaf Spinach

The small-leaf spinach seeds utilized in this experimental study were procured from Shenyang Best Agricultural Technology Co., Ltd. (Shenyang, China) The air-dried soil was treated with CdCl_2_·2.5H_2_O and equilibrated for two weeks, which was followed by two rounds of high-temperature and high-pressure sterilization (121 °C, 30 min, 1.10 MPa). After measurement according to Section 4.5.4, the treated soil contained 33.74 mg/kg of Cd and 0.31 μg/g of Se, within individual pots each filled with 3000 g of soil. The seeds were soaked overnight in distilled water, which was followed by a 1 min disinfection in 75% ethanol and a 15 min disinfection in 2.5% sodium hypochlorite [68]. Uniformly sized seeds were selected and planted in seedling trays for cultivation in an artificial climate chamber. Upon attaining the four-leaf developmental stage, seedlings of uniform size and height were relocated into individual containers, ensuring that each pot accommodated a single seedling. There were six groups in the KL19 strain experimental setup: the control group (CK), the KL19 strain treatment group, and four SeNPs treatment groups with different concentrations (5, 10, 20, 50 mg/L). To obtain the inoculum of strain KL19, the spore suspension was prepared in accordance with the procedure outlined by Ortiz et al. [69]. Five days after seedling transplantation, a one-time injection of 100 mL of spore suspension (5 × 10^7^/mL) was administered into the fungal treatment group (with no further injection), while the SeNPs treatment groups received a daily injection of 10 mL of SeNPs solution at different concentrations, which was administered once daily for a duration of seven weeks. The other groups were injected with an equal volume of sterile water instead. All spinach seedlings were grown in a controlled-environment chamber (day/night: 14/10 h; 25/18 °C; light intensity: 10,000 LX; humidity: 75%).

#### 4.5.2. Measurement of Growth Parameters and Chlorophyll Content

After seven weeks, the plants were harvested and thoroughly cleaned with distilled water. The fresh weight, leaf count, and root length of the plants in each group were recorded. The roots were severed and subjected to desiccation in an oven maintained at 65 °C for a duration of 48 h, subsequently enabling the measurement of their dry mass. To quantify the chlorophyll content in the leaves, an 80% acetone solution was employed for extraction, which was followed by spectrophotometric analysis at specific wavelengths of 645 nm, 652 nm, and 663 nm. This allowed for the determination of chlorophyll a, chlorophyll b, and the overall chlorophyll content in the leaves [70]. The remaining plant tissues were preserved at −80 °C after being treated with liquid nitrogen.

#### 4.5.3. Antioxidant Capacity Detection

The H_2_O_2_ kit (purchased from Suzhou Gris Biotechnology Co., Ltd., Suzhou, China) was utilized for the quantification of H_2_O_2_ content in the leaves, while the MDA, SOD and POD contents were quantified following the methodology outlined by Zang et al. [71].

#### 4.5.4. Cd and Se Contents Detection

The small leaf spinach seedlings were divided into two parts: underground (roots) and aboveground (stems and leaves). These sections were then subjected to dehydration in a heated chamber set at 65 °C until a stable mass was attained, indicating complete dryness. The specimens derived from both sections underwent individual pulverization processes, resulting in a meticulously refined powder that was capable of traversing a 60-mesh sieve effortlessly. Then, 0.2 g of each sample was placed in a 25 mL conical flask. In accordance with the protocol outlined by Narayanasamy et al. [72], the samples underwent digestion employing a blend of hydrochloric acid (37% mass/volume) and strong nitric acid (69% mass/volume) in a 3:1 volumetric ratio. Subsequently, the Cd content was quantified utilizing an atomic absorption spectrophotometric instrument (ZA3300, Hitachi, Tokyo, Japan), while the Se concentration was assessed with an atomic fluorescence spectrometer (PF52, Purkinje, Beijing, China). Ultimately, we determined the bioconcentration factor (BCF) and translocation factor (TF) associated with Cd [73,74].

### 4.6. Statistical Analysis

All experiments were performed in three replicates. The data were presented by mean ± standard deviation (SD). For statistical analysis, including one-way ANOVA and Duncan’s multiple range test (*p* < 0.05), the software package SPSS 27.0 was employed. We used the mapping software Origin 2021 for mapping.

## 5. Conclusions

This study highlighted the remarkable potential of an endophytic fungus *C. fructicola* and its derived SeNPs in enhancing spinach plants growth under Cd stress. The optimized SeNPs exhibited superior yield, dispersibility, and stability, facilitating their efficient permeation and utilization within spinach plants. Impressively, both KL19 and its derived 5 mg/L SeNPs significantly alleviated Cd-induced toxicity by boosting photosynthesis, increasing biomass, and modulating antioxidant defenses to mitigate oxidative stress. Furthermore, they may impede Cd uptake and accumulation by altering plasma membrane permeability or sequestering Cd into less bioavailable forms. In conclusion, these findings underscored the promising application of *C. fructicola* KL19 and its SeNPs in mitigating Cd stress, offering a novel approach to promote vegetable productivity and safeguard edible plant parts from harmful Cd contamination in polluted soils.

## Figures and Tables

**Table 1:** Independent variables quantity planned by BBD.

Factor	Low Level (−1)	Medium Level (0)	High Level (+1)
Fresh weight (g)	1	2	3
Concentration of Na_2_SeO_3_ (mM)	2	4.5	7
pH	6	7	8

**Table 2:** Predicted and actual production of SeNPs under optimal parameter values.

Fresh Weight (g)	Na_2_SeO_3_ (mM)	pH	Se^0^ (Pre)	Se^0^ (Act)
2.62	4.56	6.25	1.02	1.06

## Data Availability

All data are contained within the article.

## Supplementary Materials

The following supporting information can be downloaded at https://www.mdpi.com/article/10.3390/plants13172359/s1, Figure S1: Growth of 20 strains on PDA with 2 mM Na_2_SeO_3_, the red color indicates that the strain reduced selenite to elemental red selenium (Se^0^); Figure S2: Tolerance test of strain KL19 to Cd and Se; Tabel S1: The 20 genera of endophytic fungus both in *Kadsura angustifolia* and *Schisandra sphenanthera*; Table S2: Box–Behnken design of experiment matrix and experimental results for yields of Se^0^; Table S3: Variance analysis (ANOVA) for the BBD focusing on Se^0^ content.
