# Supplementary material for: Endophytic Colletotrichum fructicola KL19 and Its Derived SeNPs Mitigate Cd-Stress-Associated Damages in Spinacia oleracea L
Source: Plants (Basel). 2024 Aug 24;13(17):2359. doi: 10.3390/plants13172359 (PMC11396860; doi:10.3390/plants13172359)
Supplement: Supplementary file 1 [file plants-13-02359-s001.zip › Supplementary Materials 1.pdf]

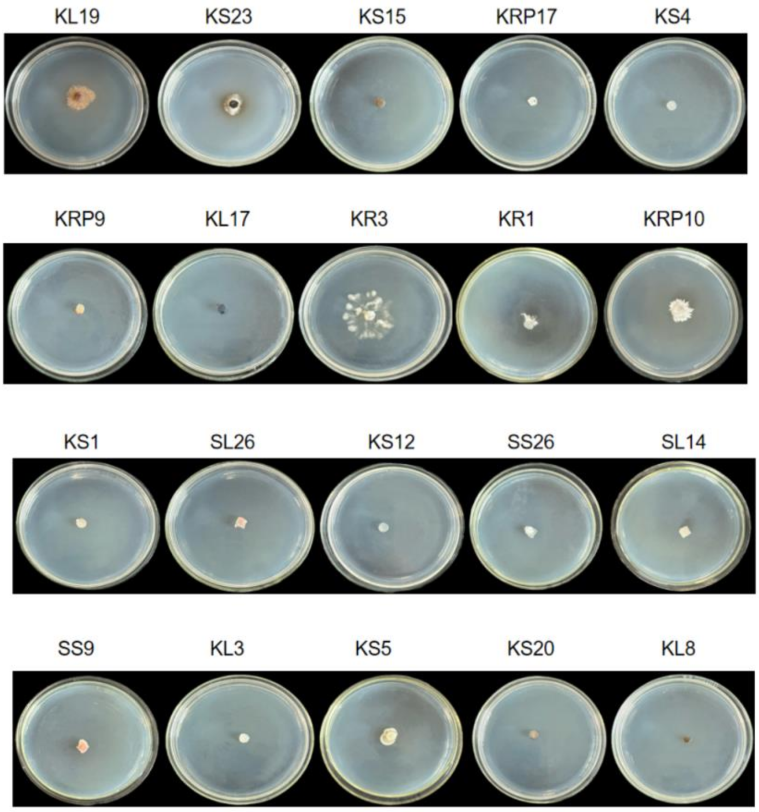

Figure S1. Growth of 20 strains on PDA with 2mM Na_2SeO_3. The red color indicates that the strain reduced selenite to elemental red selenium (Se^0)

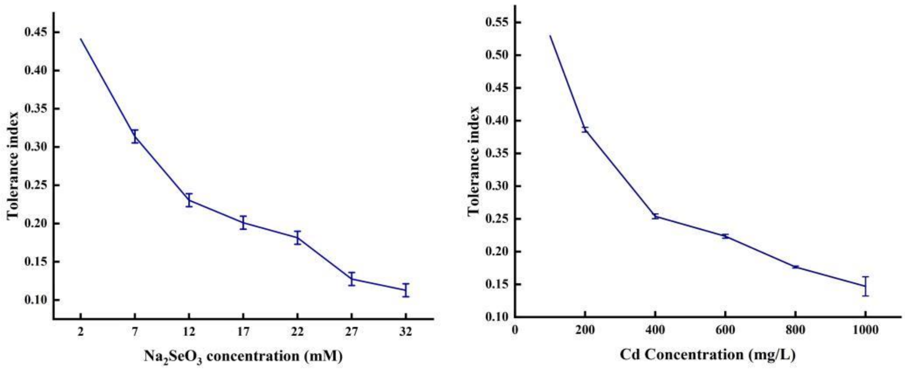

Figure S2. Tolerance Test of Strain KL19 to Cd and Se
