# Supplementary material for: Endophytic Colletotrichum fructicola KL19 and Its Derived SeNPs Mitigate Cd-Stress-Associated Damages in Spinacia oleracea L
Source: Plants (Basel). 2024 Aug 24;13(17):2359. doi: 10.3390/plants13172359 (PMC11396860; doi:10.3390/plants13172359)
Supplement: Supplementary file 1 [file plants-13-02359-s001.zip › Supplementary Materials 2.pdf]

Table S1 20 genera of endophytic fungus both in *Kadsura angustifolia* and *Schisandra sphenanthera.*

numbers	
KL19	*Colletotrichum fructicola*
KS23	*Hypoxylon investiens*
KS15	*Diaporthe discoidispora*
KRP17	*Clitopilus prunulus*
KS4	*Diaporthe vaccinii*
KRP9	*Ceratobasidium sp.*
KL17	*Guignardia mangiferae*
KR3/KRP10	*Neopestalotiopsis sp.*
KR1	*Pestalotiopsis microspora*
KS1	*Daldinia eschscholtzii*
SL26	*Colletotrichum acutata*
KS12	*Phomopsis sp.*
SS26	*Mucor irregularis*
SL14	*Colletotrichum boninense*
SS9	*Emmia lacerata*
KL3	*Eutypella scoparia*
KS5	*Trichoderma hamatum*
KS20	*Botryosphaeria dothidea*
KL8	*Beltrania rhombica*

Table S2 Box-Behnken design of experiment matrix and experimental results for yields of Se^0.

Run	A:Fresh weight (g)	B:Na_2SeO_3 (mM)	C:pH	Response1 Se^0 (μmol/mL)
1	1	2	7	0.4294
2	2	7	6	1.0496
3	1	4.5	8	0.5308
4	2	2	6	0.9155
5	2	4.5	7	0.7583
6	2	2	8	0.6908
7	2	4.5	7	0.8066
8	2	4.5	7	0.8233
9	1	7	7	0.8293
10	3	4.5	6	1.1295
11	2	4.5	7	0.7746
12	3	2	7	1.1951
13	2	7	8	0.9110
14	3	4.5	8	1.1279
15	1	4.5	6	0.7140
16	2	4.5	7	0.8694
17	3	7	7	0.8896

Table S3 Variance analysis (ANOVA) for the BBD focusing on Se^0 content.

Source	Sum of Squares	df	Mean Square	F-value	P-value
Model	0.6198	6	0.1033	23.36	<0.0001
A:Fresh weight	0.4226	1	0.4226	95.56	<0.0001
B:Na2SeO3	0.0252	1	0.0252	5.69	0.0382
C:pH	0.0376	1	0.0376	8.49	0.0155
AB	0.1244	1	0.1244	28.13	0.0003
AC	0.0082	1	0.0082	1.86	0.2020
BC	0.0019	1	0.0019	0.4191	0.5320
Residual	0.0442	10	0.0044		
Lack of Fit	0.0366	6	0.0061	3.22	0.1386
Pure Error	0.0076	4	0.0019		
Cor Total	0.6640	16			
Adjusted R2	0.8934				
R2	0.9334				
Std.Dev.	0.0665				
C.V.%	7.83				
